# Correspondence

**Published:** 1906-05

**Authors:** Roswell Park

**Affiliations:** Buffalo


### CORRESPONDENCE.

*Dr. Roswell Park castigates The Physicians Publishing Company.—He did not write for their for their book.—He repudiates the statements they make in the most emphatic manner.*

Editor Buffalo Medical Journal.

Sir:—Can you find space in the next issue of the Journal for the enclosed letter? I would like to publish it to prevent further imposition so far as I can. These men have taken advantage of my name to sell a number of books and I want to repudiate the statements they have made, in the most emphatic and public manner. If you can give it a place, you will oblige me and perhaps protect others.

Roswell Park.

Buffalo, April 16, 1906.

Physicians Publishing Company,
1211 Filbert St.,
Philadelphia, Pa.
Also 95 Milk St.,
Boston, Mass.

Gentlemen: It has come to my knowledge that a book has been recently sold by house to house canvass in this city, being presented especially to nurses, having some such title as The Household Physician and Twentieth Century Materia Medica, of which you purport to be the publishers. On the title page is a statement in which the names of several eminent authors in the country are used, as well as my own, indicating that the book is compiled from our writings. Much worse than this, your agents who have been selling it in this city have used my own name in an absolutely dishonest and lying manner, saying that I wrote especially for the work, and making other statements even more strong, inducing those with whom I was personally acquainted to buy it upon such dishonest representation.

The whole method is a contemptible and disreputable attempt to misuse names and mislead purchasers, which I wish to rebuke as publicly as I can, and especially to protest against the use of my name in any such way as that in which in has been used. I shall call the attention of the police to the fact, and I desire to inform you that I shall protect myself in every possible way against any such unwarranted and deceptive use of my name.

The method of its use is bad enough, but to be made responsible for the misleading and entirely wrong statements made within the book is still worse. I warn you, therefore, to discontinue your practice and to instruct your agents accordingly, since I shall resort to every expedient which the law permits to prevent further fraud and deception in the premises.

Buffalo, N. Y. April 16, 1906. ROSWELL PARK.

